# Oral amoxicillin for management of fast breathing or chest indrawing pneumonia without danger signs in children aged 2–59 months: a systematic review and meta-analysis

**DOI:** 10.7189/jogh.16.04209

**Published:** 2026-09-11

**Authors:** Barsha Gadapani Pathak, Yasir Bin Nisar, Uma Chandra Mouli Natchu, Rukman Manapurath, Archana Thakur, Pranay Vats, Temsunaro Rongsen Chandola, Bireshwar Sinha

**Affiliations:** 1 Society for Applied Studies, New Delhi, India; 2 Centre for Intervention Science in Maternal and Child Health, Centre for International Health, University of Bergen, Department of Global Public Health and Primary Care, Bergen, Norway; 3 Department of Sexual, Reproductive, Maternal, Child and Adolescent Health and Ageing (LHR), World Health Organization (WHO), Geneva, Switzerland; 4 Department of Biotechnology/Wellcome India Alliance Clinical and Public Health Fellow, Hyderabad, India

**Keywords:** under-five children, fast breathing pneumonia, chest-indrawing pneumonia, oral amoxicillin, injectable antibiotics, systematic review and meta-analysis

## Abstract

**Background:**

World Health Organization (WHO) guidelines recommend oral amoxicillin as first-line therapy for pneumonia in children aged 2–59 months. However, uncertainty remains regarding its effectiveness compared with no antibiotics for fast breathing pneumonia and with injectable antibiotics for chest indrawing pneumonia without danger signs.

**Methods:**

We searched PubMed, EMBASE, and the Cochrane Central Register of Controlled Trials (January 2000–August 2024) for randomised and quasi-randomised trials without language restrictions. Eligible studies compared oral amoxicillin *vs.* no antibiotics for fast breathing pneumonia or outpatient oral amoxicillin *vs.* inpatient injectable antibiotics for chest indrawing pneumonia without danger signs in children (2–59 months). Pooled relative risks (RRs) with 95% confidence intervals (CIs) were estimated using a random-effects model; certainty was assessed using GRADE.

**Results:**

Four trials (n = 7,699) compared oral amoxicillin with no antibiotics for fast breathing pneumonia, and three (n = 4,864) compared oral with injectable antibiotics for chest indrawing pneumonia. For fast breathing pneumonia, oral amoxicillin reduced treatment failure by 16% at day 14 (RR = 0.84; 95% CI = 0.75, 0.94, *I*^2^ = 0%; moderate certainty). For chest indrawing pneumonia, treatment failure was similar between groups at day 6 (RR = 0.96; 95% CI = 0.83, 1.11) and day 14 (RR = 0.94; 95% CI = 0.79, 1.13), while mortality was 72% lower with oral amoxicillin (RR = 0.28; 95% CI = 0.09, 0.86). Serious adverse events did not differ between groups.

**Conclusions:**

In children (2–59 months), oral amoxicillin significantly reduces treatment failure compared to no antibiotics for fast breathing pneumonia and is comparable to injectable antibiotics for chest indrawing pneumonia without danger signs. Notably, oral amoxicillin was associated with a 72% reduction in mortality compared with injectable antibiotics in children with chest-indrawing pneumonia, although absolute risk differences were small. These findings support its broader implementation as an accessible, programmatically feasible, and WHO-recommended option for pneumonia management in low-resource settings.

**Registration:**

PROSPERO: CRD42023439851

Pneumonia remains a leading cause of under-five mortality in low- and middle-income countries (LMICs) despite progress in child health over the past two decades [1,2]. Alongside other major causes of child mortality, such as prematurity, intrapartum-related complications, diarrhoea, and malaria, pneumonia continues to account for a substantial proportion of preventable under-five deaths globally [3]. Achieving Sustainable Development Goal 3, which aims to end preventable child deaths by 2030, will therefore require strengthened prevention and management of major childhood illnesses, particularly pneumonia, in high-burden regions such as sub-Saharan Africa and South Asia, where healthcare access remains limited [4]. To address childhood pneumonia management in resource-limited settings, the World Health Organization (WHO) has established evidence-based recommendations within the Integrated Management of Childhood Illness (IMCI) framework [5]. According to the WHO 2014 pneumonia management guidelines, children aged 2–59 months with fast breathing pneumonia without chest indrawing or any general danger signs like inability to drink or breastfeed, persistent vomiting, convulsions, lethargy or unconsciousness, should be treated with oral amoxicillin (40 mg/kg/dose twice daily) for five days with home-based supportive care [6]. In settings with low HIV prevalence, a three-day course of amoxicillin is recommended for treating fast breathing pneumonia in children, based on evidence demonstrating comparable clinical cure rates and no significant differences in treatment failure compared to a five-day regimen. For children with chest indrawing pneumonia in the absence of any general danger signs, the guidelines recommend outpatient management with oral amoxicillin for five days, without the need for hospitalisation or injectable antibiotics [6]. Children with any general danger signs should be referred for inpatient care and treated with injectable antibiotics with supporting care [6]. This stratified approach is particularly relevant in LMICs, where hospital access and diagnostic capacity are often constrained [7,8].

The optimal management of fast breathing and chest indrawing pneumonia in children under five remains a subject of ongoing research. Under-five deaths from bacterial pneumonia have declined markedly since 1990, driven by improved access to antibiotics and the introduction of Hib and pneumococcal vaccines in national immunisation programme [4]. Evidence from a review and a study conducted in seven African and Asian countries showed that viral aetiology accounts for around 60% of all hospitalised cases of chest indrawing pneumonia with or without danger signs in children aged 1–59 months [8,9]. Given this evidence, along with concerns about antimicrobial resistance, the necessity of antibiotics for all children with fast breathing pneumonia is questioned [6,10–13]. Despite these shifts in the epidemiology of childhood pneumonia, current treatment guidelines continue to recommend universal antibiotic therapy for WHO-defined fast breathing pneumonia. This creates an important clinical and policy tension: while antibiotics are effective in reducing treatment failure, a substantial proportion of pneumonia cases may be viral and self-limiting, raising concerns about unnecessary antibiotic exposure and antimicrobial resistance. At the same time, withholding antibiotics in resource-limited settings, where diagnostic uncertainty is high, may increase the risk of adverse outcomes. This uncertainty highlights the need to re-examine the balance between benefits and risks of antibiotic use considering evolving evidence.

In 2021, a Cochrane review showed that for the treatment of fast breathing pneumonia in children aged 2–59 months, use of oral amoxicillin compared to placebo or no antibiotics was associated with a 20% reduction in treatment failure [1]. Consistently, a recent meta-analysis of 13 LMIC studies also found higher treatment failure with placebo compared to amoxicillin (OR = 1.40; 95% CI = 1.00, 1.96), supporting continued antibiotic use for WHO-defined fast breathing pneumonia [14]. Although the 2021 Cochrane review synthesised available evidence on antibiotic use for fast breathing pneumonia, it did not incorporate more recent trials conducted in diverse LMIC settings nor fully reflect updated WHO classifications and programmatic contexts [15]. With evolving epidemiology and increasing recognition of viral aetiology, newer studies may provide important insights to strengthen treatment recommendations. For chest indrawing pneumonia, key uncertainties remain regarding the need for hospitalisation and parenteral antibiotics *vs.* WHO-recommended outpatient oral amoxicillin [16]. While earlier reviews have assessed the efficacy of oral antibiotics for severe pneumonia, a specific gap remains in directly comparing oral amoxicillin with injectable antibiotics for chest indrawing pneumonia, as defined by the latest WHO guidelines [17]. Although, three trials have been conducted comparing oral Amoxicillin *vs.* injectable antibiotics to treat children with chest indrawing pneumonia [18–20], their findings have not been synthesised in the context of updated WHO recommendations.

Given the persisting uncertainties and new evidence, an updated review is warranted to inform current clinical practice. Specifically, this review re-examines the effectiveness of antibiotic strategies within the context of evolving epidemiology, increasing recognition of viral aetiology, and updated WHO classifications. We therefore aimed, first, to synthesise the evidence on the efficacy of oral amoxicillin compared to no antibiotic treatment in children aged 2–59 months with fast breathing pneumonia (without chest indrawing or general danger signs), with treatment failure as the primary outcome. Second, to assess the effect of oral amoxicillin administered on an outpatient basis *vs.* inpatient injectable antibiotics for children presenting with chest indrawing pneumonia without danger signs. This review incorporates recent trials aligned with the current WHO pneumonia classifications and provides a synthesis of up-to-date evidence that can inform future WHO guidelines on the management of childhood pneumonia.

## METHODS

We conducted a comprehensive search in the Cochrane Central Register of Controlled Trials (CENTRAL), Cochrane Register of Studies Online, PubMed®, and EMBASE® databases to identify relevant articles on randomised controlled trials or quasi-randomised trials published before 31 August 2024. We targeted finding trials that compared oral amoxicillin with either no antibiotic treatment or placebo in children with fast breathing pneumonia aged 2–59 months, or trials that compared outpatient oral amoxicillin to inpatient injectable antibiotics for chest indrawing pneumonia in children aged 2–59 months. The search strategies are outlined in Box S1 in the **Online Supplementary Document**. We did not impose any language restrictions in our search, but we limited the search dates from the year 2000 to August 2024. This date restriction was implemented because trials conducted before 2000 may not align with the current management protocols outlined in WHO guidelines. Articles published in languages other than English were reviewed, and data were extracted from English abstracts when available, or translated using an online application if needed. Additionally, we manually searched the reference lists of selected articles to identify further relevant studies. This review is registered in the PROSPERO prospective register of systematic reviews (CRD42023439851) and follows the Preferred Reporting Items for Systematic Reviews and Meta-Analyses (PRISMA) 2020 statement.

### Outcomes

The primary outcomes studied were treatment failure among children with fast breathing or chest indrawing pneumonia. For the first question (oral amoxicillin compared to no antibiotic treatment in children with fast breathing pneumonia), the definition of treatment failure (after three days of treatment) included mortality, persistence of fast breathing (as per the WHO criteria), or appearance of chest indrawing, or presence of any general danger signs, or need for higher treatment (*e.g.* need to add another antibiotic, or change to a higher antibiotic, or hospital admission). We have assessed treatment failure at day four and day 14 from treatment initiation. For the second question (oral amoxicillin administered on an outpatient basis *vs.* inpatient injectable antibiotics in children presenting with chest-indrawing pneumonia) the definition of treatment failure (after five days of treatment) included mortality, persistence of chest indrawing, or appearance of any general danger signs, or need for higher treatment (*e.g.* need to add another antibiotic, or change to a higher antibiotic, or hospital admission). We have assessed treatment failure at day six and day 14 from treatment initiation. Given the variability in definitions of treatment failure across included trials, we adopted a harmonised outcome definition aligned with WHO clinical criteria, incorporating persistence or progression of symptoms, development of danger signs, or need for treatment escalation. Where study-specific definitions differed, outcomes were mapped to this common framework to ensure comparability across studies. We used the treatment failure definitions as reported by the original study authors and aligned these conceptually with this framework without reclassifying individual participant data. The definitions used by each study included in this systematic review are detailed in the Box S2 and S3 in the **Online Supplementary Document****.**

Secondary outcomes in both the study questions comprised mortality, serious adverse events (SAEs), and cost-effectiveness. Serious adverse events included symptoms like nausea, vomiting, diarrhoea, rash, itching, tremors, mouth ulcers, severe diarrhoea necessitating intravenous hydration, anaphylaxis, and organ failure. Secondary outcomes were reported at the most recent follow-up assessment. Mortality was defined as all-cause death occurring during the study follow-up period (up to day 14 from initiation of treatment), irrespective of attribution to pneumonia or other causes.

### Study selection and data collection

We utilised the Covidence systematic review software from Veritas Health Innovation in Melbourne, Australia, to review the data [21]. Two authors independently screened titles and abstracts to identify relevant citations based on predefined inclusion criteria. Full-text reviews were then conducted for selected articles. Data extraction was performed using a modified version of the Cochrane Effective Practice and Organization of Care (EPOC) group data collection checklist from the Cochrane EPOC Group in London, UK and Northern Ireland [22]. This checklist included details such as study identifiers, study context, design, intervention specifics, dose and duration of treatment, and study outcomes. Any disagreements or discrepancies between reviewers were resolved through discussion or by involving a third author for review.

### Data analysis

We conducted the data analysis in accordance with the guidelines outlined in the Cochrane Handbook for Systematic Reviews of Interventions [22]. Stata, version 16 (StataCorp LLC in College Station, TX, USA) was used for the analysis. Pooled relative risks (RRs) along with 95% confidence intervals (CIs) were reported for categorical variables [23]. We employed a random-effects model with the restricted maximum likelihood method to combine data and estimate effects, as the included studies were conducted across different settings and periods [23]. This approach accounts for both within-study and between-study variability, making it appropriate given the expected methodological differences and variations in implementation across the included studies. Egger’s test was employed to assess publication bias for outcomes reported in at least five studies [23].

We evaluated the risk of bias in the included studies by using the revised Cochrane risk-of-bias tool for randomised trials (RoB 2, London, UK) [22,24]. We assessed the certainty of the evidence for our pooled outcome estimates using the GRADE approach [25]. PRISMA guidelines were used to report the findings [26]. Furthermore, we conducted a post-hoc analysis by pooling studies conducted after 2015, following the rollout of influenza vaccination, to examine its impact on all primary and secondary outcomes.

## RESULTS

For the first research question (oral amoxicillin compared to no antibiotic treatment in children with fast breathing pneumonia), our initial database search on 31 August 2024 identified 6,645 records (Figure S1 in the **Online Supplementary Document**). After removing duplicates and screening titles and abstracts, 62 articles were selected for full-text review. Four relevant randomised controlled trials from three countries were included in our meta-analysis (**Table 1**) that enrolled a total of 7,699 children aged 2–59 months with fast breathing pneumonia [15,27–29]. Of the included trials, three had a low risk of bias [15,27,28], and one trial had a high risk of bias [29] (Figure S2 in the **Online Supplementary Document**). Three trials were conducted in low- or lower-middle-income countries [15,27,28] and one was conducted in low-income countries [29]. The control group of fast breathing pneumonia among 2–59 months old children received no antibiotics or placebo. The trials that were conducted before 2015, *i.e*. Awasthi *et al*. and Hazir *et al*. had given amoxicillin dosages ranging from 15 mg/kg/d to 54 mg/kg/d every eight hours for three days [27,28], and the trials after that, *i.e*. Ginsburg *et al*. and Jehan *et al*. gave oral amoxicillin based on weight bands which ranged from 50 mg/kg to 75 mg/kg/d for three days [15,29]. There was one trial providing oral amoxicillin at the primary level [15] and three trials providing care at the secondary or tertiary level [27–29]. Two trials were conducted before the roll-out of the pneumococcal conjugate vaccination [27,28].

**Table 1:** Description of studies of oral amoxicillin *vs*. no antibiotics/placebo among children aged 2–59 months with fast-breathing pneumonia

Author, study design, country, settings	Population	Intervention	Control	Sample size (n)	Outcome	Effect size (95% CI)
Awasthi, 2008, RCT, India, hospital-based (tertiary)	2–59 months WHO case definition for fast breathing pneumonia	Oral amoxicillin (31–54 mg/kg/d, 8 hourly for 3 days)	No antibiotic	1,671	Primary: treatment failure by day 3. Secondary: clinical cure and relapse.	Risk difference 4.2% in favour of antibiotic, 95% CI = 0.2, 8.1
Hazir, 2011, RCT, Pakistan, hospital-based (tertiary)	2–59 months WHO case definition for fast breathing pneumonia	Oral amoxicillin (15 mg/kg/d, 8 hourly for 3 days)	No antibiotic	900	Primary: treatment failure at 3 days. Secondary: relapse at 14 days.	OR = 0.85; 95% CI = 0.50, 1.43, *P* = 0.60).
Ginsburg, 2019, RCT, Malawi, tertiary care centres	2–59 months WHO case definition for fast breathing pneumonia	Oral amoxicillin, 500 mg/d for 2–11 months, 1000 mg/d for 12–35 months, 1500 mg/d for 36–59 months for 3 days	No antibiotics	1,343	Treatment failure at 4 and 14 days. Secondary: relapse 5–14 days, adverse events.	RR = 1.16; 95% CI = 0.83%, 1.63% at 14 days
Jehan, 2020, RCT, Pakistan, primary care centres	2–59 months WHO case definition for fast breathing pneumonia	Oral amoxicillin, 500 mg every 12 h for a weight of 4 to <10 kg, 1000 mg every 12 h for a weight of 10 to <14 kg, 1500 mg every 12 h for a weight of 14 to <20 kg for 3 days	No antibiotics	4,002	Treatment failure at 3 and 14 days. Secondary: adverse events.	Risk-difference = 2.3 percentage points; 95% CI = 0.9, 3.7

CI – confidence interval, OR – odds ratio, RR – risk ratio

For the second research question, oral amoxicillin administered on an outpatient basis *vs.* inpatient injectable antibiotics in children presenting with chest-indrawing pneumonia, our initial database search on 31 August 2024 identified 6645 records. After removal of duplicates, screening titles and abstracts, and full-text review, three randomised controlled trials were included [18–20] that enrolled a total of 4864 children aged 2–59 months with chest indrawing pneumonia (**Table 2**; Figure S3 in the **Online Supplementary Document**). There were some concerns of bias in all three trials (Figure S4 in the **Online Supplementary Document****)** [18–20]. These trials represented nine countries; one was a multicentre trial (eight sites from LMIC settings in Asia, Africa, and Latin America), one in Pakistan, and the other in Kenya. Children with chest indrawing pneumonia in the control group received injectable ampicillin or penicillin. In the children in the intervention group, amoxicillin dosages ranged from 45 mg/kg to 90 mg/kg twice daily for 5 days. As most pooled analyses included fewer than five studies, formal assessment of publication bias was not feasible for all outcomes.

**Table 2:** Description of the studies conducted on oral amoxicillin *vs.* injectable antibiotics: among children aged 2–59 months with chest indrawing pneumonia.

Author, study design, country	Population	Intervention	Control	Sample size	Outcome	Effect size (95% CI) risk difference %
Addo-Yobo, RCT, 2004, (Columbia, South Africa, Vietnam, Pakistan, Ghana, Mexico, India, Zambia)	2–59 months WHO case definition for chest indrawing pneumonia and/or fast breathing	Oral amoxicillin 45mg/kg/d in three doses for 5 days	Injectables (inj. penicillin)	1,702	Primary: treatment failure by day 2. Secondary: treatment failure by day 5 and 14.	Risk difference: −0.4%; (95% CI −4.2, 3.3) at 2 days
Hazir, RCT, Pakistan, 2008	2–59 months WHO case definition for chest indrawing pneumonia and/or fast breathing	Oral amoxicillin, 80–90 mg/kg per day in two doses for 5 days	Injectable ampicillin	2,037	Primary: treatment failure by day 2. Secondary: treatment failure by day 5 and 14.	Risk difference: 1.1%; (95% CI = −1.3, 3.5) at 6 days
Agweyu, RCT, Kenya, 2015	2–59 months WHO case definition for chest indrawing pneumonia and/or fast breathing	Oral amoxicillin, 40–45 mg/kg twice daily for 5 days	Injectable penicillin	527	Primary: treatment failure by day 2. Secondary: treatment failure by day 5 and 14.	Risk difference = −0.3% (95% CI = −5.0%, 4.3%) at 2 days

CI – confidence interval

### Primary outcomes

#### Oral antibiotics *vs.* no antibiotics/placebo in children with fast breathing pneumonia

**Figure 1:**
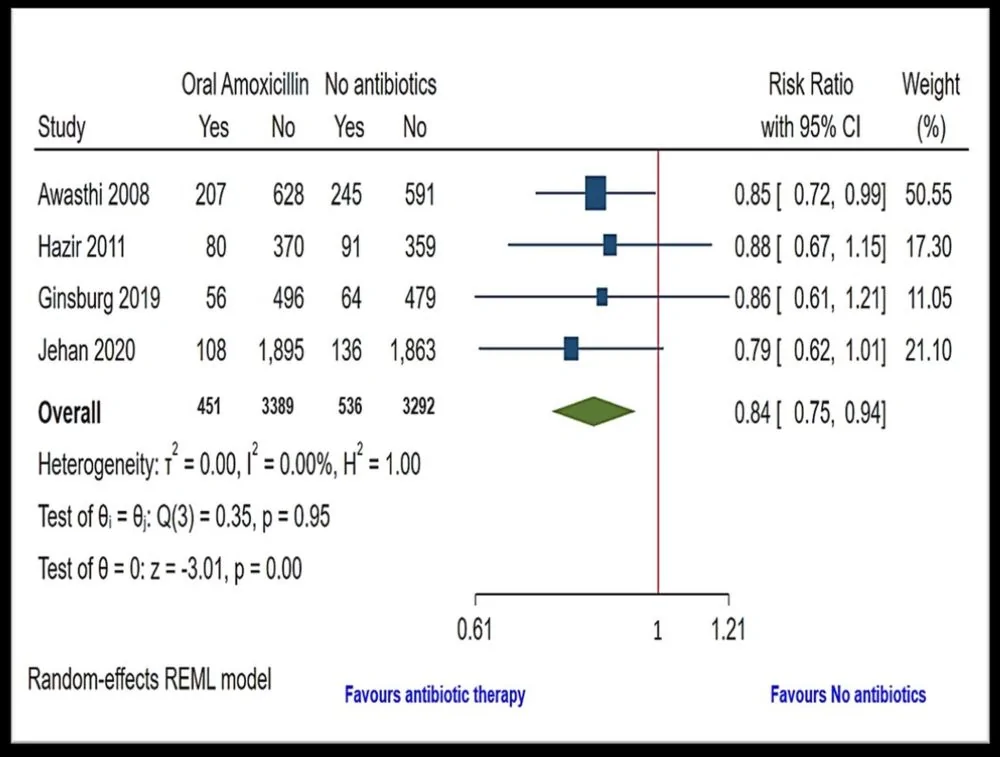
Oral antibiotics *vs.* no antibiotics/placebo: risk of treatment failure in children (2–59 months) with fast breathing pneumonia assessed at 14 days. CI – confidence interval.

In children with fast breathing pneumonia treated with oral amoxicillin compared with children who received a placebo or no antibiotics, the pooled RR of treatment failure (including mortality) assessed at four days from treatment initiation was 0.74 (95% CI = 0.53, 1.02, *I*^2^ = 74.11%), based on four trials involving 7,668 participants, with low certainty evidence (Figure S5 in the **Online Supplementary Document**). At 14 days from treatment initiation, the risk of treatment failure was 16% lower in children treated with oral amoxicillin compared with children who received a placebo or no antibiotics (RR = 0.84; 95% CI = 0.75, 0.94, *I*^2^ = 0.00%), based on four trials involving 7,668 participants, with moderate certainty evidence (**Figure 1**).

#### Oral antibiotics *vs.* injectables in children with chest indrawing pneumonia

**Figure 2:**
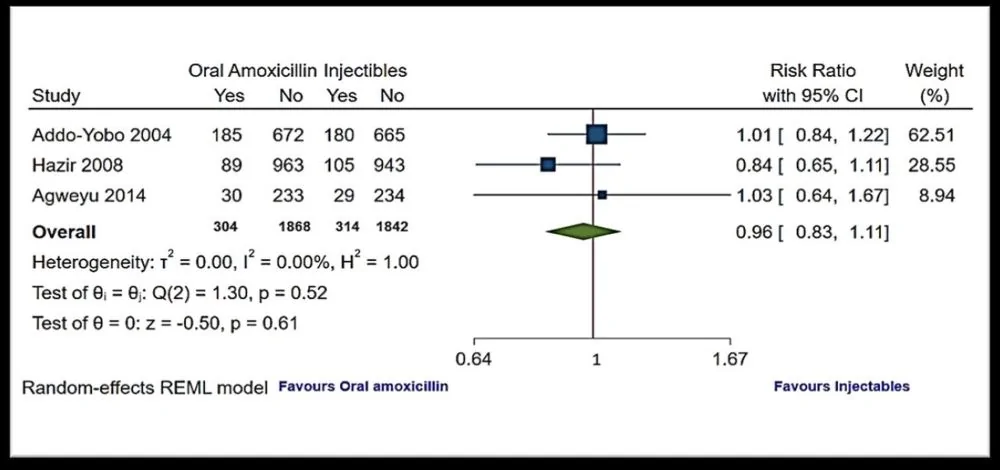
Oral amoxicillin *vs.* injectable antibiotics: treatment failure among children (2–59 months) with chest indrawing pneumonia on the 6th day of management. CI – confidence interval.

**Figure 3:**
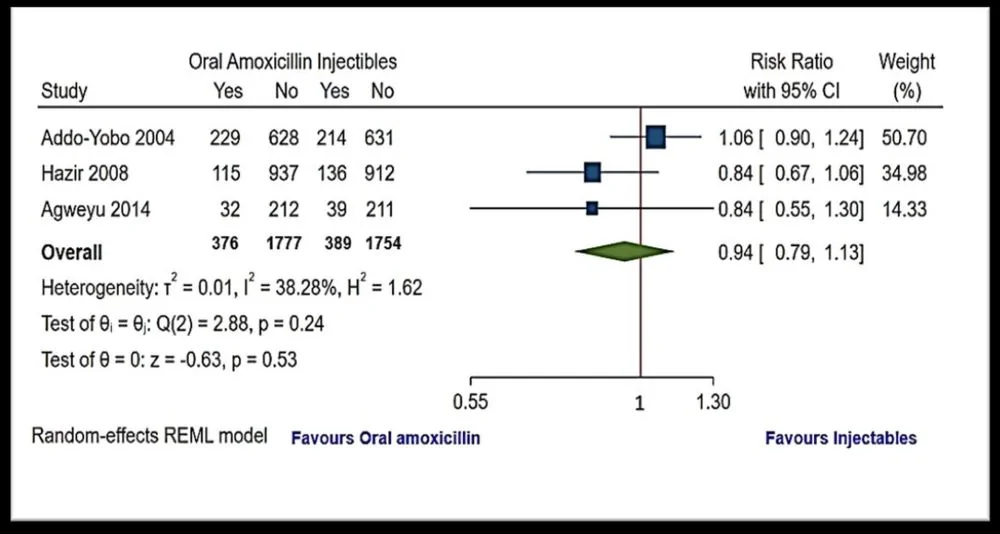
Oral amoxicillin *vs.* injectable antibiotics: treatment failure among children (2–59 months) with chest indrawing pneumonia on the 14th day from treatment initiation. CI – confidence interval.

In children with chest indrawing pneumonia managed with oral amoxicillin at outpatient compared with those who received inpatient injectable antibiotics, the pooled RR of treatment failure assessed at the sixth day from treatment initiation was RR 0.96 (95% CI = 0.83, 1.11; *I*^2^ = 0.00%) based on three trials involving 4,328 participants, with low certainty evidence) (**Figure 2**). Similarly, the pooled RR of treatment failure assessed at 14 days from treatment initiation was RR 0.94 (95% CI = 0.79, 1.1, *I*^2^ = 38.28%) based on three trials involving 4,296 participants, with low certainty evidence (**Figure 3**).

Overall, while treatment failure rates were comparable between groups, the certainty of evidence ranged from moderate (for fast-breathing pneumonia at day 14) to low (for most other outcomes), and findings should be interpreted with caution. Although definitions of treatment failure varied across studies, these were largely centred on clinically meaningful deterioration (*e.g.* persistence or worsening of symptoms, development of danger signs, or need for treatment escalation), which supports the comparability of pooled estimates. Treatment failure among children aged 2–59 months with chest -indrawing pneumonia on 3rd day of management is detailed in Figure S6 in the **Online Supplementary Document****.**

### Secondary outcomes

#### Oral antibiotics *vs.* no antibiotics/placebo in children with fast breathing pneumonia

In children with fast breathing pneumonia treated with oral amoxicillin compared with children who received a placebo or no antibiotics, the risk of any adverse events at 14 days from the initiation of treatment was similar across the groups (pooled RR = 0.93; 95% CI = 0.75, 1.15, *I*^2^ = 0.00%) based on two trials involving 5,128 participants, with low certainty evidence (Figure S7 in the **Online Supplementary Document**). Two other trials were excluded from this analysis because cumulative adverse events at 14 days were not reported, and therefore, data were not available for quantitative pooling [27,28]. The pooled risk of all-cause mortality at 14 days from treatment initiation was similar between children treated with oral amoxicillin and those receiving placebo or no antibiotics (pooled RR = 1.00; 95% CI = 0.17, 5.75, *I*^2^ = 0.00%) based on four trials involving 7,668 participants, with low certainty evidence) (Figure S8 in the **Online Supplementary Document****)**. However, this estimate is based on a very small number of events and should be interpreted with caution.

#### Oral antibiotics *vs.* injectables in children with chest indrawing pneumonia

In children with chest indrawing pneumonia managed with oral amoxicillin at outpatient compared with those who received inpatient injectable antibiotics, the pooled RR of any adverse events at 14 days from treatment initiation was 0.39 (95% CI = 0.12, 1.26, *I*^2^ = 0.00%) based on three trials involving 4,329 participants with low certainty evidence (Figure S9 in the **Online Supplementary Document**). The risk of all-cause mortality at 14 days from treatment initiation was lower among children treated with oral amoxicillin compared with those receiving inpatient injectable antibiotics (pooled RR = 0.28; 95% CI = 0.09, 0.86, *I*^2^ = 0.00%) based on three trials involving 4,329 participants, with low certainty evidence. However, this estimate is based on a small number of events and should be interpreted with caution (Figure S10 in the **Online Supplementary Document****)**. The absolute risk of mortality was 0.18% (n/N = 4/2,173) in the oral amoxicillin group compared to 0.65% (n/N = 14/2,156) in the injectable group, yielding an absolute risk difference of −0.47% (approximately 4–5 fewer deaths per 1,000 children treated). The number needed to treat (NNT) to prevent one death with oral amoxicillin instead of injectables was approximately 215 (range = 169–1100 across the 95% CI of the pooled RR).

The GRADES assessment tables for both the research questions are detailed in Tables S1 and S2 in the **Online Supplementary Document**. Additionally, two studies from Africa and Asia [15,29] carried out after the introduction of pneumococcal conjugate and H. influenzae vaccines, showing 46% (RR = 0.54; 95% CI = 0.41, 0.72) and 18% (RR = 0.82; 95% CI = 0.67, 0.99), significant reduction in treatment failure at day four and day 14, respectively, among the amoxicillin group compared to the no antibiotic group Figures S11 and S12 in the **Online Supplementary Document****.**

None of the included studies conducted formal cost-effectiveness analyses. However, three studies reported cost-related information. Hazir *et al*. [15] highlighted potential reductions in costs associated with outpatient management, while Jehan *et al*. [20] discussed the broader economic implications of antibiotic use at individual and societal levels. Awasthi *et al*. [27] provided a crude cost estimate, suggesting that treating 24 children with oral amoxicillin would avert one treatment failure at an approximate cost of 7.2 USD, based on drug costs. These findings represent limited and heterogeneous economic evidence and cannot be interpreted as formal cost-effectiveness.

## DISCUSSION

Our systematic review included four trials on fast breathing pneumonia and three on chest indrawing pneumonia in children aged 2–59 months across LMICs. For fast breathing pneumonia, treatment with oral amoxicillin was associated with a modest but statistically significant reduction in treatment failure by day 14 compared with no antibiotic use, supported by moderate-certainty evidence, whereas the effect observed at day four was less precise and based on low-certainty evidence. For chest indrawing pneumonia, outpatient oral amoxicillin showed treatment failure rates comparable to inpatient injectable antibiotics at days six and 14, based on low to very low certainty evidence. Meta-analyses also suggest lower mortality and similar risks of any adverse events with oral amoxicillin *vs.* injectables, although this finding is based on a small number of events and low-certainty evidence, supporting oral amoxicillin as a viable option for managing chest-indrawing pneumonia without danger signs.

Our findings align with and expand upon previous research, including the 2021 Cochrane review, which observed a 20% reduction in treatment failure when using oral amoxicillin compared to placebo in children under five with fast breathing pneumonia [1]. In comparison, our review includes one additional trial published after the Cochrane 2021 review, thereby providing updated evidence. Our review, alongside the findings from other systematic analyses, underscores the continuing importance of oral amoxicillin in managing fast breathing pneumonia, particularly in LMICs where healthcare, hospitalisation, and diagnostic capacity are often limited [14]. Our review adds to the evidence base by incorporating studies published after 2015, allowing evaluation of oral amoxicillin’s efficacy within the context of updated WHO guidelines and expanded PCV and Hib immunisation [15,29]. Notably, the studies by Ginsburg *et al*. (2019) and Jehan *et al*. (2020) conducted in populations with widespread pneumococcal conjugate and *Haemophilus influenzae* type b vaccination, indicate that oral amoxicillin remains more effective than no antibiotics for fast breathing pneumonia, even as the proportion of viral pneumonia appears to be increasing [15,29]. With widespread vaccination against Streptococcus pneumoniae and *Haemophilus influenzae* type B, bacterial pneumonia rates have declined, and the relative proportion of viral pneumonias is reported to have increased [13,30]. Despite this change in pathogen profile, our findings indicate that oral amoxicillin remains effective. This sustained efficacy may be attributed to the presence of residual bacterial infections or bacterial-viral co-infections, which are not uncommon in children presenting with fast breathing pneumonia [11,31]. Such co-infections can worsen disease severity and may respond to antibiotics, as suggested by the reduced treatment failure in pooled analyses. Hence, oral amoxicillin remains an essential tool for treating pneumonia where bacterial involvement cannot be ruled out, highlighting its role in empirical treatment approaches that prioritise feasibility in resource-limited settings. The substantial heterogeneity observed may be attributable to differences in study settings (primary *vs.* hospital-based care), variations in antibiotic dosing regimens, and, to a lesser extent, differences in outcome definitions across studies.

The efficacy of oral amoxicillin compared with injectable antibiotics in treating chest indrawing pneumonia is further supported by earlier reviews, such as those by Rojas-Reyes *et al.*, Lodha *et al.*, and Das *et al.*, which found no significant differences in treatment failure rates between oral and injectable regimens at key assessment points [8,17,32]. Our pooled findings add to this body of evidence by showing comparable outcomes between the oral and injectable groups, while also identifying a lower mortality risk in the oral group. While the pooled relative estimate suggested a reduction in mortality favouring oral amoxicillin, the absolute risk of death was low in both groups (0.18% *vs.* 0.65%), corresponding to an absolute risk reduction of 0.47% *i.e*. approximately 4–5 fewer deaths per 1,000 children and a number needed to treat of approximately 215. These findings highlight that the apparent relative benefit translates into a modest absolute effect. Importantly, the estimate is based on a small number of events (18 deaths across three trials), with wide confidence intervals and low-certainty evidence, and should therefore be interpreted cautiously. All included trials were open-label, raising the possibility of differential surveillance and outcome ascertainment. In addition, causes of death were heterogeneous and often unrelated to pneumonia-specific treatment effects. Taken together, while the direction of effect is reassuring, mortality differences alone should not be considered definitive evidence for policy decisions.

These findings suggest that outpatient oral therapy can be a safe and effective alternative, particularly where hospitalisation is difficult to access or imposes high costs on families. The pharmacokinetic properties of amoxicillin allow it to reach therapeutic levels in the bloodstream and respiratory epithelial lining fluid, effectively targeting bacterial pathogens like Streptococcus pneumoniae without requiring invasive administration methods [33]. This clinical non-inferiority between oral and injectable routes has important policy implications, offering an opportunity to safely reduce hospitalisation burden in LMICs while expanding access to treatment [8]. The use of oral amoxicillin reduces reliance on hospital-based care, lowering costs, minimising injection-related risks, and increasing accessibility, especially in remote areas where access to healthcare facilities may be a barrier to timely treatment [34].

Nonetheless, we acknowledge the limitations of low certainty evidence, and the findings should be interpreted with caution and locally contextualised. Recent prospective cohort studies from Uganda, Nigeria, Zambia, Ethiopia, and Pakistan conducted in routine programme settings report low case fatality rates (generally <1%), low hospitalisation rates, and high clinical recovery among children with chest indrawing pneumonia managed with outpatient oral amoxicillin. These real-world findings complement trial evidence and suggest the feasibility and safety of outpatient oral amoxicillin under routine IMCI implementation in LMICs [35–39].

Notably, outcome definitions varied across included trials, with ‘treatment failure’ ranging from persistent symptoms to the development of danger signs or need for hospitalisation. Such heterogeneity may partly explain variability in effect estimates; however, the core components of treatment failure definitions were clinically similar across studies, suggesting that the direction of effect is unlikely to be substantially altered. At the same time, while oral amoxicillin offers a practical alternative to injectable antibiotics, outpatient oral management may also reduce treatment costs for families and health systems, decrease unnecessary hospital admissions, and lower the risk of healthcare-associated infections (HAIs), which remain a concern in resource-limited hospital settings. Recent programme-based cohort studies further highlight these system-level benefits of outpatient care [35,37–39].

While our review did not assess antimicrobial resistance outcomes directly, the potential implications of widespread antibiotic use remain important. Expanded use highlights the need for careful antibiotic stewardship to prevent antimicrobial resistance (AMR) [40–44]. Clear diagnostic criteria are essential to ensure that amoxicillin use is reserved for bacterial cases, minimising unnecessary antibiotic exposure in cases where viral pathogens are clearly responsible [34]. Therefore, these considerations should be interpreted as broader public health implications rather than findings directly derived from this review. Also, some studies reported cost-related information, including crude estimates and qualitative economic implications, none performed formal cost-effectiveness analyses. Therefore, conclusions regarding cost-effectiveness cannot be drawn from the available evidence. These gaps warrant further experimental studies on cost-effectiveness and antimicrobial resistance. Ongoing implementation research in real-world low resource settings, may also provide valuable evidence on costs, feasibility, community and health system implications of oral amoxicillin use [45–48].

### Strengths and limitations

A key strength of our meta-analysis is its inclusion of recent studies, which enables us to assess the relevance of oral amoxicillin within updated WHO classifications and post-vaccine contexts. Additionally, our focus on low-resource settings provides contextually significant insights that can inform pneumonia management strategies and policymaking in LMICs. We conducted a comprehensive search across multiple databases, resulting in robust inclusion of relevant studies. Collectively, the trials enrolled 12,532 participants, 7668 for oral amoxicillin *vs.* no antibiotics for fast breathing pneumonia, and 4864 for oral amoxicillin *vs.* injectables for chest indrawing pneumonia (with no danger signs) in 2–59-month-old children, offering substantial statistical power to evaluate both primary and secondary outcomes, with rigorous quality assessment conducted using the GRADE approach to enhance the reliability of the pooled evidence.

However, there are limitations to consider. On ROB assessment, all trials included for evaluating the effectiveness of oral amoxicillin *vs.* injectables presented ‘some concerns’, and one trial included for the question of oral amoxicillin *vs.* no antibiotics had a high risk of bias, which affected the certainty of our conclusions. Furthermore, the certainty of evidence was predominantly low to very low, particularly for chest-indrawing pneumonia outcomes, due to these design inconsistencies and limited sample sizes for certain outcomes. Importantly, this limited certainty reduces confidence in the observed effects and warrants cautious interpretation, especially for outcomes such as mortality, where estimates are based on a small number of events. Future studies employing uniform methodologies and harmonised outcome definitions across diverse settings will be essential to confirm these findings and strengthen the recommendations for pneumonia management in under-resourced areas. The absence of formal economic evaluations, including data on caregiver costs and health system burden, limits the ability to assess the comparative cost-effectiveness of outpatient *vs.* inpatient treatment strategies.

## CONCLUSIONS

Our review supports the use of oral amoxicillin as more effective than no antibiotics for treating fast breathing pneumonia and as a potentially safe and effective alternative to injectable antibiotics for chest indrawing pneumonia without danger signs in children aged 2–59 months. These findings highlight the value of oral amoxicillin for wider implementation in LMICs, where healthcare resources are constrained as an accessible and programmatically feasible treatment option. Future research should aim to refine diagnostic criteria to guide antibiotic use more precisely, thereby improving pneumonia management while minimising the risk of antimicrobial resistance.

## Additional material


Online Supplementary Document

## Data Availability

**Data availability:** All the data utilised for the analysis is presented in this manuscript. Any further query could be addressed to the corresponding author.
