# Supplementary material for: Oral amoxicillin for management of fast breathing or chest indrawing pneumonia without danger signs in children aged 2–59 months: a systematic review and meta-analysis
Source: J Glob Health. 2026 Sep 11;16:04209. doi: 10.7189/jogh.16.04209 (PMC13563637; doi:10.7189/jogh.16.04209)
Supplement: Supplementary file 1 [file jogh-16-04209-s001.pdf]

Supplement to: Gadapani Pathak B, Nisar YB, Natchu UCM, Manapurath R, Thakur A, Vats P, Chandola TR, Sinha B. Oral amoxicillin for management of fast breathing or chest indrawing pneumonia without danger signs in children aged 2–59 months: a systematic review and meta-analysis. J Glob Health. 2026;16:04209.

Barsha Gadapani Pathak, [1,2] (0000-0003-4574-580X) Yasir Bin Nisar,[3] (0000-0002-9720-5699) Uma Chandra Mouli Natchu,[1] Rukman Manapurath, [1,2] Archana Thakur,[1] Pranay Vats,[1] Temsunaro Rongsen Chandola,[1] Bireshwar Sinha,[1,4*] (0000-0002-7408-7474)

[1] Society for Applied Studies, New Delhi, India

[2] Centre for Intervention Science in Maternal and Child Health, Centre for International Health, University of Bergen, Department of Global Public Health and Primary Care, Bergen, Norway

[3]Department of Sexual, Reproductive, Maternal, Child and Adolescent Health and Ageing (LHR), World Health Organization (WHO), Geneva, Switzerland

[*]**Corresponding author**

Bireshwar Sinha, PhD, Society for Applied Studies. 45, Kalu Sarai, New Delhi-110016, India, E-mail: bireshwar.sinha@sas.org.in

Content

Supplementary Figure 1: PRISMA flowchart for oral amoxicillin versus no antibiotics/placebo among children 2-59 months old with fast breathing pneumonia.

Supplementary Box 1: Search strategy for research questions: Oral Amoxicillin versus Placebo and Oral Amoxicillin versus Injectable antibiotics

Supplementary Figure 2: Risk of bias for oral amoxicillin versus no antibiotics for treating fast breathing pneumonia among 2-59 months old children.

Supplementary Figure 3: PRISMA flowchart for oral amoxicillin versus injectables among children 2-59 months old with chest indrawing pneumonia

Supplementary Figure 4: Risk of bias for oral amoxicillin versus injectables for treating chest indrawing pneumonia among 2-59 months old children.

Supplementary Figure 5: Risk of treatment failure in children (2-59 months) with fast breathing pneumonia assessed at 4 days.

Supplementary Table 1: In children 2-59 months of age with only fast breathing, what is the effectiveness of oral amoxicillin compared to no antibiotic treatment in improving clinical outcomes.

Supplementary Table 2: In children 2-59 months of age with chest indrawing (without any danger signs), what is the effectiveness of oral amoxicillin on an outpatient basis compared to inpatient injectable antibiotics treatment in improving clinical outcomes.

Supplementary Figure 6: Oral Amoxicillin versus Injectable antibiotics: Treatment failure among children aged 2-59 months with chest -indrawing pneumonia on 3rd day of management.

Supplementary Figure 7: Oral Amoxicillin versus Injectable antibiotics: Adverse events among children aged 2-59 months with chest indrawing pneumonia on the 14th day from treatment initiation.

Supplementary Figure 8: Risk of all-cause mortality among the 2-59 months old children managed for fast breathing pneumonia

Supplementary Figure 9: Oral Amoxicillin versus Injectable antibiotics: Adverse events among children aged 2-59 months with chest indrawing pneumonia on the 14th day from treatment initiation.

Supplementary Figure 10: Oral Amoxicillin versus Injectables antibiotics: All-cause mortality among children managed for chest indrawing pneumonia on the 14th day.

Supplementary Figure 11: Post hoc analysis following the role-out of influenzas vaccine: Risk of treatment failure in children (2-59 months) with fast-breathing pneumonia assessed at 4 days

Supplementary Figure 12: Post hoc analysis following the role-out of influenzas vaccine: Risk of treatment failure in children (2-59 months) with fast-breathing pneumonia assessed at 14 days

Supplementary Box 1 and Box 3: Operational definitions.

Supplementary Figure 1: PRISMA flowchart for oral amoxicillin versus no antibiotics/placebo among children 2-59 months old with fast breathing pneumonia.

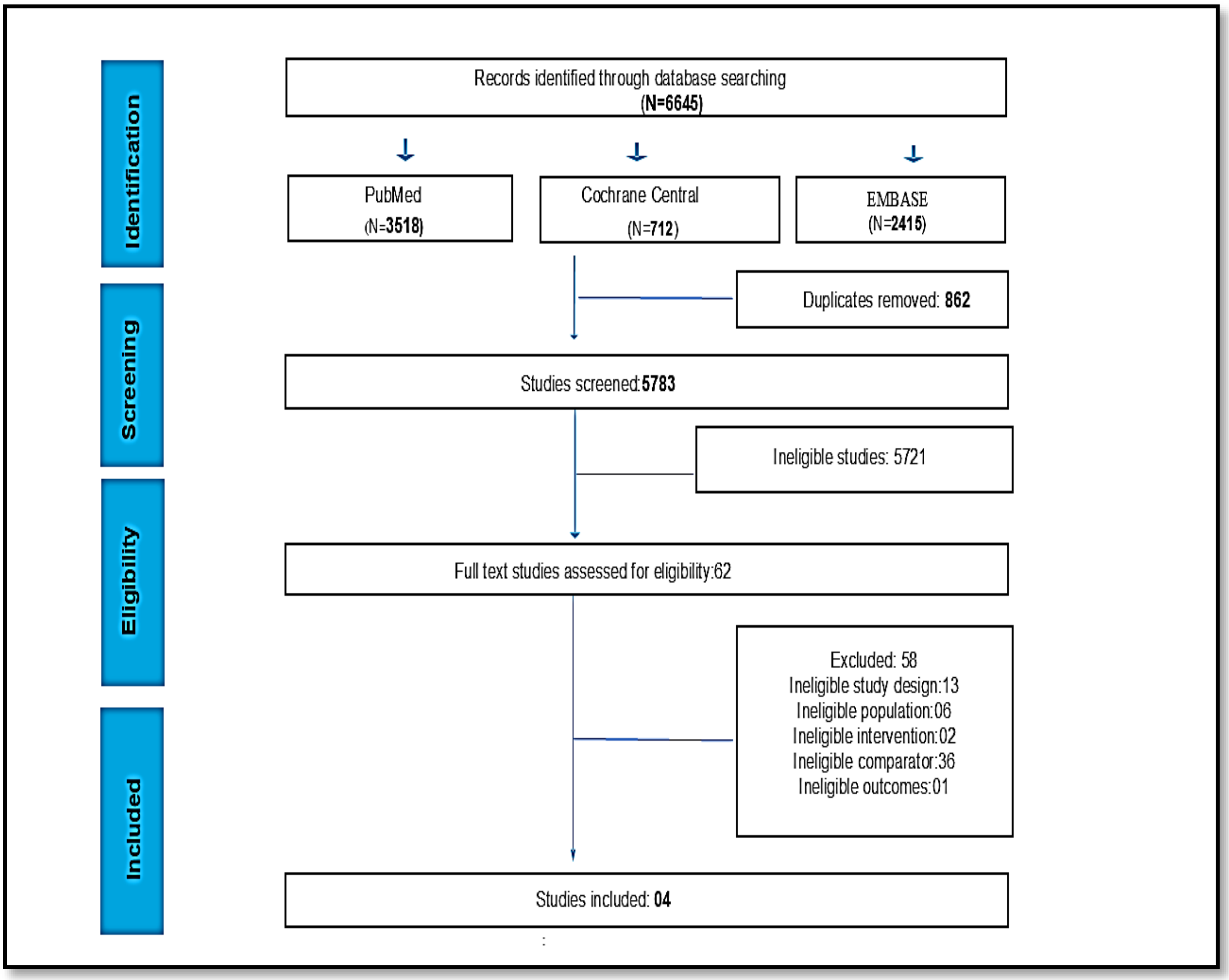

Supplementary Box 1: Search strategy for research questions: Oral Amoxicillin versus Placebo and Oral Amoxicillin versus Injectable antibiotics

**PubMed search strategy (Oral Amoxicillin versus No antibiotics)**		
**Search block**	Terms	Hits
**#1 (Disease)**	("pneumonia"[All Fields]) OR (pneumon*[tw]) OR ("bronchopneumonia"[All Fields]) OR ("pleuropneumonia"[All Fields]) OR ("fast breathing pneumonia"[All Fields]) OR (difficult breathing [tiab]) OR ("tachypnea"[All Fields]) OR ("chest indrawing pneumonia"[All Fields]) OR ("bronchiolitis"[All Fields])	358357
**#2 (Population, only 0-5 years)**	(((((((((Infant[MeSH Terms]) OR (infant*[Title/Abstract])) OR (infancy[Title/Abstract])) OR (newborn*[Title/Abstract])) OR (baby*[Title/Abstract])) OR (babies[Title/Abstract])) OR (neonat*[Title/Abstract])) OR (preterm*[Title/Abstract])) OR	2954091

	(prematur*[Title/Abstract])) OR (child*[Title/Abstract])) OR ("under-five"[All Fields]) OR ("under"[All Fields] AND "five*"[All Fields]) OR (preschool*[Title/Abstract])	
**#3 (Medicines)**	(((((((((((Antibiotic*[MeSH Terms]) OR (Anti-bacterial agent*[MeSH Terms])) OR (Anti-infective agent*[MeSH Terms])) OR (Penicillins [MeSH Terms])) OR (antibiotics[MeSH Terms])) OR (antibacterial*[Text Word])) OR (anti-bacterial[Text Word])) OR (bacteriocid*[Text Word])) OR (antimicrobial[Text Word])) OR (antimicrobial*[Text Word])) OR (antiinfective*[Text Word])) OR (anti-infective*[Text Word])) OR (amoxicillin[MeSH Terms])	1074788
**#4 (Study Design)**	("cohort studies"[MeSH Terms] OR "cross sectional studies"[MeSH Terms] OR "randomized controlled trial"[Publication Type] OR "controlled clinical trial"[Publication Type] OR "clinical trial"[Publication Type] OR "Randomized"[Title/Abstract] OR "placebo"[Title/Abstract] OR "clinical trials as topic"[MeSH Major Topic] OR "randomly"[Title/Abstract] OR "Trial"[Title] OR "non randomized controlled trial*"[MeSH Major Topic] OR "case control studies"[MeSH Terms] OR "observational studies"[Text Word] OR "observational studies"[Title/Abstract] OR "Preprint"[Publication Type])	4506272
**#5 (Publication types)**	(Address*[ptyp] OR Autobiography[ptyp] OR Bibliography[ptyp] OR Biography[ptyp] OR pubmed books[filter] OR Case Reports[ptyp] OR Congress*[ptyp] OR Consensus Development Conference[ptyp] OR Directory[ptyp] OR Duplicate Publication[ptyp] OR Editorial[ptyp] OR Festschrift[ptyp] OR Guideline[ptyp] OR Interview[ptyp] OR Lecture*[ptyp] OR Legal Case*[ptyp] OR News[ptyp] OR Newspaper Article[ptyp] OR Personal Narrative*[ptyp] OR Portrait*[ptyp] OR Retracted Publication[ptyp] OR Twin Study[ptyp] OR Video-Audio Media[ptyp])	3685995
**#6 Search block for Q3 and Q4**	# 1 AND # 2 AND # 3 AND #4	3673
**#7 (FINAL FOR 3 &4)**	#6 NOT #5, Filter humans ONLY	3518

**PubMed search strategy for RQ 4 (Oral Amoxicillin versus Injectable antibiotics)**		
**Search block**	Terms	Hits
**#1 (Disease)**	("pneumonia"[All Fields]) OR (pneumon*[tw]) OR ("bronchopneumonia"[All Fields]) OR ("pleuropneumonia"[All Fields]) OR ("fast breathing pneumonia"[All Fields]) OR (difficult breathing [tiab]) OR ("tachypnea"[All Fields]) OR ("chest indrawing pneumonia"[All Fields]) OR ("bronchiolitis"[All Fields])	358357

**#2 (Population, only 0-5 years)**	(((((((((Infant[MeSH Terms]) OR (infant*[Title/Abstract])) OR (infancy[Title/Abstract])) OR (newborn*[Title/Abstract])) OR (baby*[Title/Abstract])) OR (babies[Title/Abstract])) OR (neonat*[Title/Abstract])) OR (preterm*[Title/Abstract])) OR (prematur*[Title/Abstract])) OR (child*[Title/Abstract])) OR ("under-five"[All Fields]) OR ("under"[All Fields] AND "five*"[All Fields]) OR (preschool*[Title/Abstract])	2954091
**#3 (Medicines)**	(((((((((((Antibiotic*[MeSH Terms]) OR (Anti-bacterial agent*[MeSH Terms])) OR (Anti-infective agent*[MeSH Terms])) OR (Penicillins [MeSH Terms])) OR (antibiotics[MeSH Terms])) OR (antibacterial*[Text Word])) OR (anti-bacterial[Text Word])) OR (bacteriocid*[Text Word])) OR (antimicrobial[Text Word])) OR (antimicrobial*[Text Word])) OR (antiinfective*[Text Word])) OR (anti-infective*[Text Word])) OR (amoxicillin[MeSH Terms])	1074788
**#4 (Study Design)**	("cohort studies"[MeSH Terms] OR "cross sectional studies"[MeSH Terms] OR "randomized controlled trial"[Publication Type] OR "controlled clinical trial"[Publication Type] OR "clinical trial"[Publication Type] OR "Randomized"[Title/Abstract] OR "placebo"[Title/Abstract] OR "clinical trials as topic"[MeSH Major Topic] OR "randomly"[Title/Abstract] OR "Trial"[Title] OR "non randomized controlled trial*"[MeSH Major Topic] OR "case control studies"[MeSH Terms] OR "observational studies"[Text Word] OR "observational studies"[Title/Abstract] OR "Preprint"[Publication Type])	4506272
**#5 (Publication types)**	(Address*[ptyp] OR Autobiography[ptyp] OR Bibliography[ptyp] OR Biography[ptyp] OR pubmed books[filter] OR Case Reports[ptyp] OR Congress*[ptyp] OR Consensus Development Conference[ptyp] OR Directory[ptyp] OR Duplicate Publication[ptyp] OR Editorial[ptyp] OR Festschrift[ptyp] OR Guideline[ptyp] OR Interview[ptyp] OR Lecture*[ptyp] OR Legal Case*[ptyp] OR News[ptyp] OR Newspaper Article[ptyp] OR Personal Narrative*[ptyp] OR Portrait*[ptyp] OR Retracted Publication[ptyp] OR Twin Study[ptyp] OR Video-Audio Media[ptyp])	3685995
**#6 Search block for Q3 and Q4**	# 1 AND # 2 AND # 3 AND #4	3673
**#7** (FINAL FOR **3 &4)**	#6 NOT #5, Filter humans ONLY	3518

Supplementary Figure 2: Risk of bias for oral amoxicillin versus no antibiotics for treating fast breathing pneumonia among 2-59 months old children.

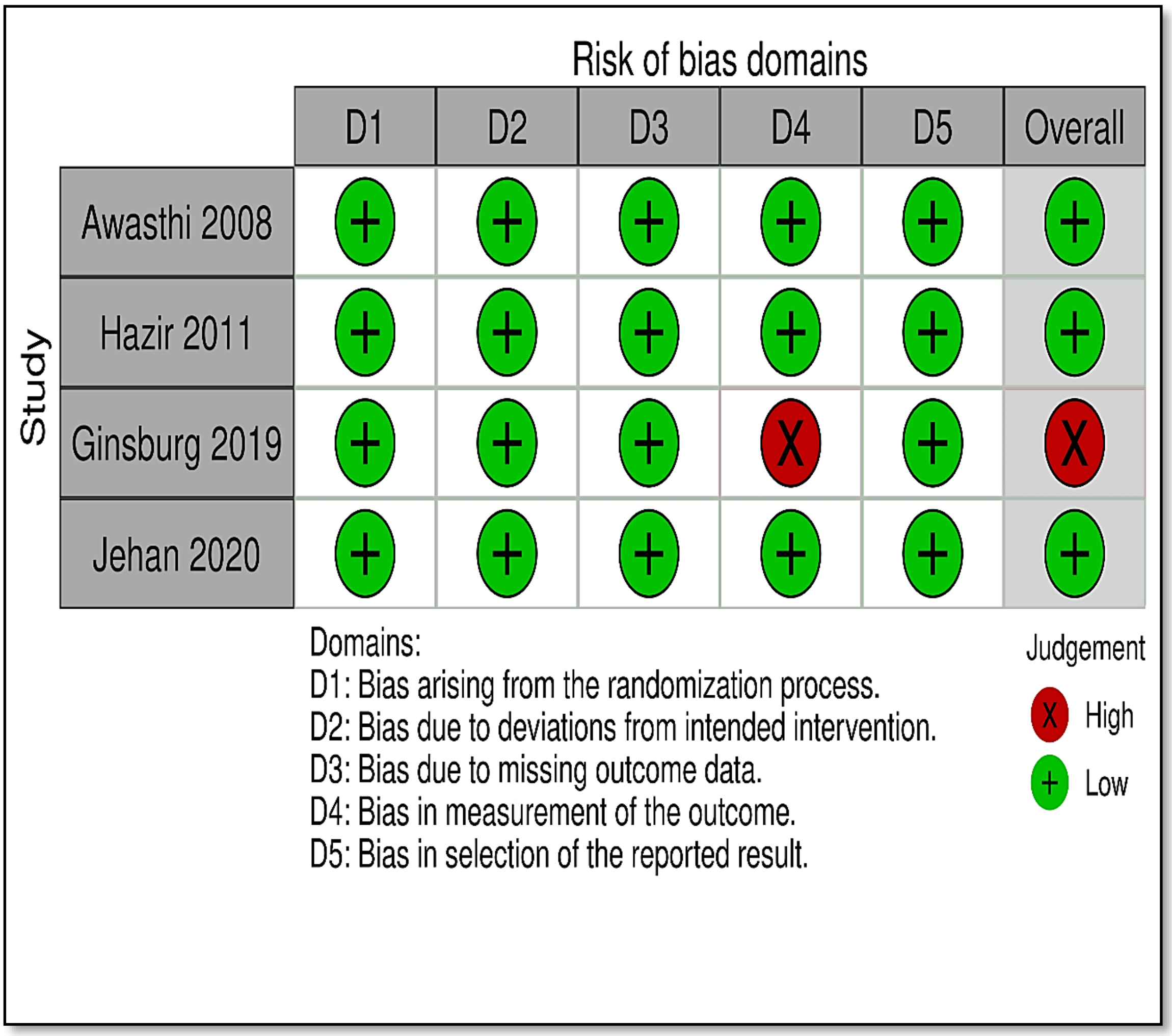

Supplementary Figure 3: PRISMA flowchart for oral amoxicillin versus injectables among children 2-59 months old with chest indrawing pneumonia

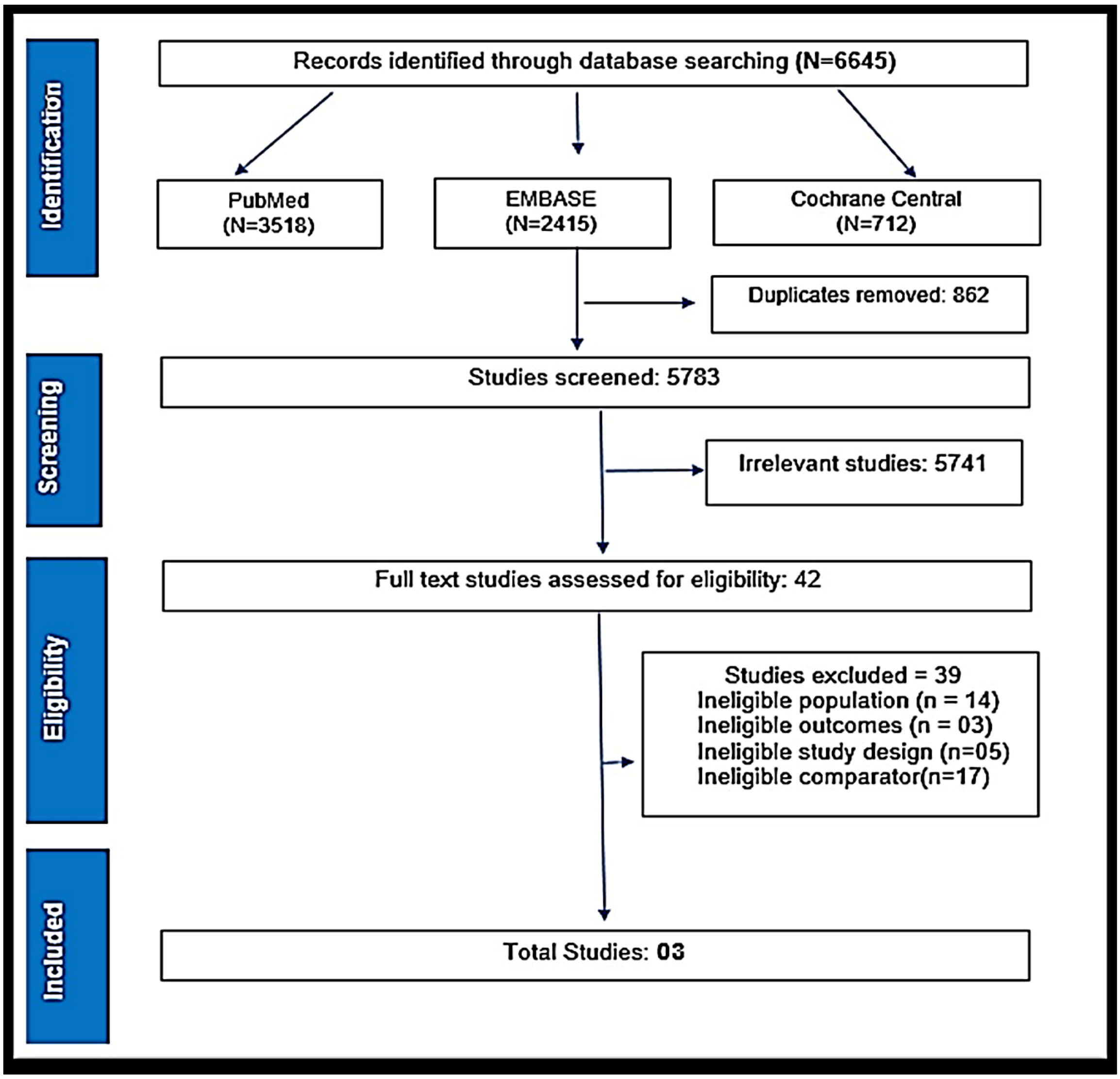

Supplementary Figure 4: Risk of bias for oral amoxicillin versus injectables for treating chest indrawing pneumonia among 2-59 months old children.

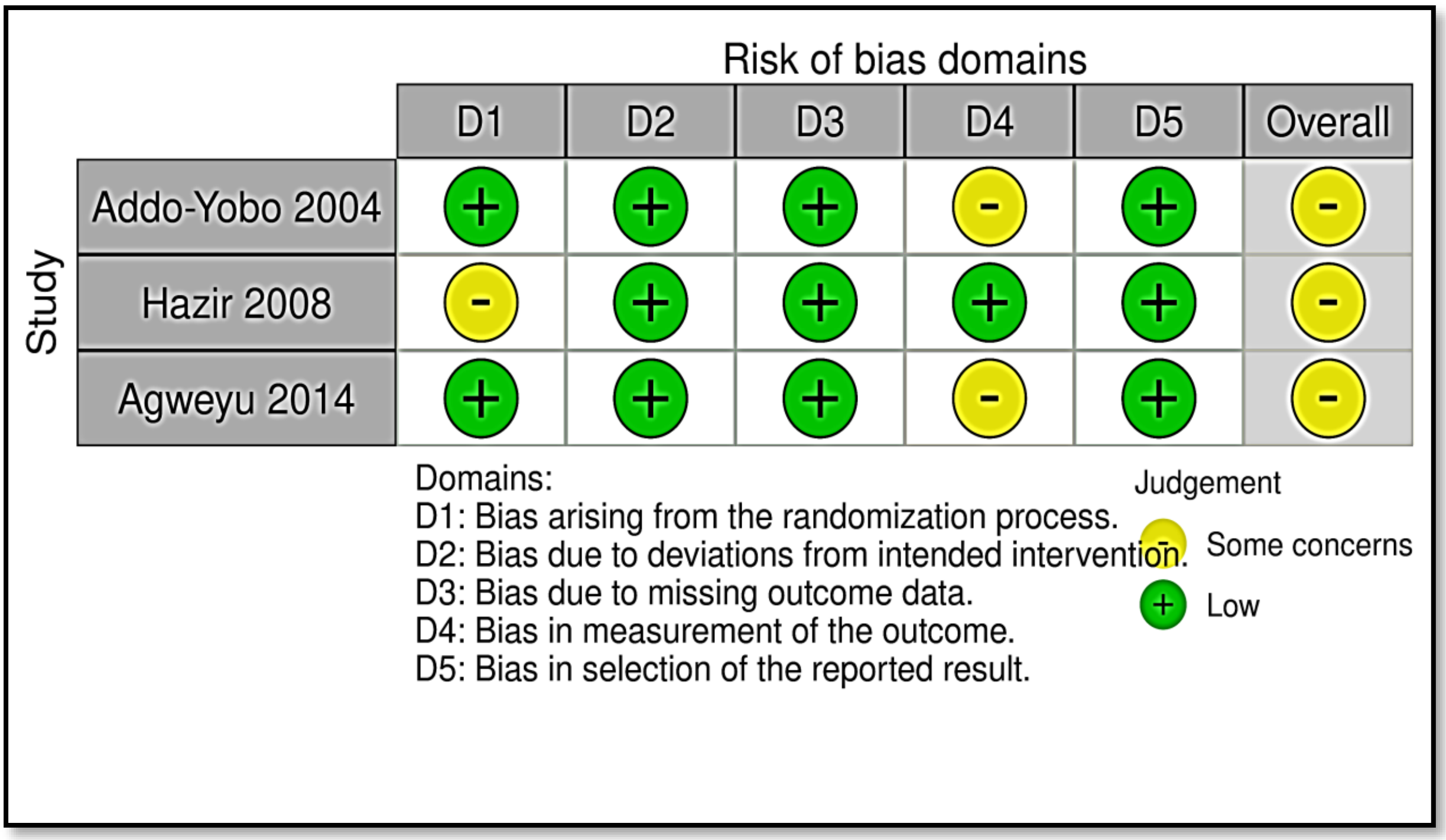

Supplementary Figure 5: Risk of treatment failure in children (2-59 months) with fast breathing pneumonia assessed at 4 days

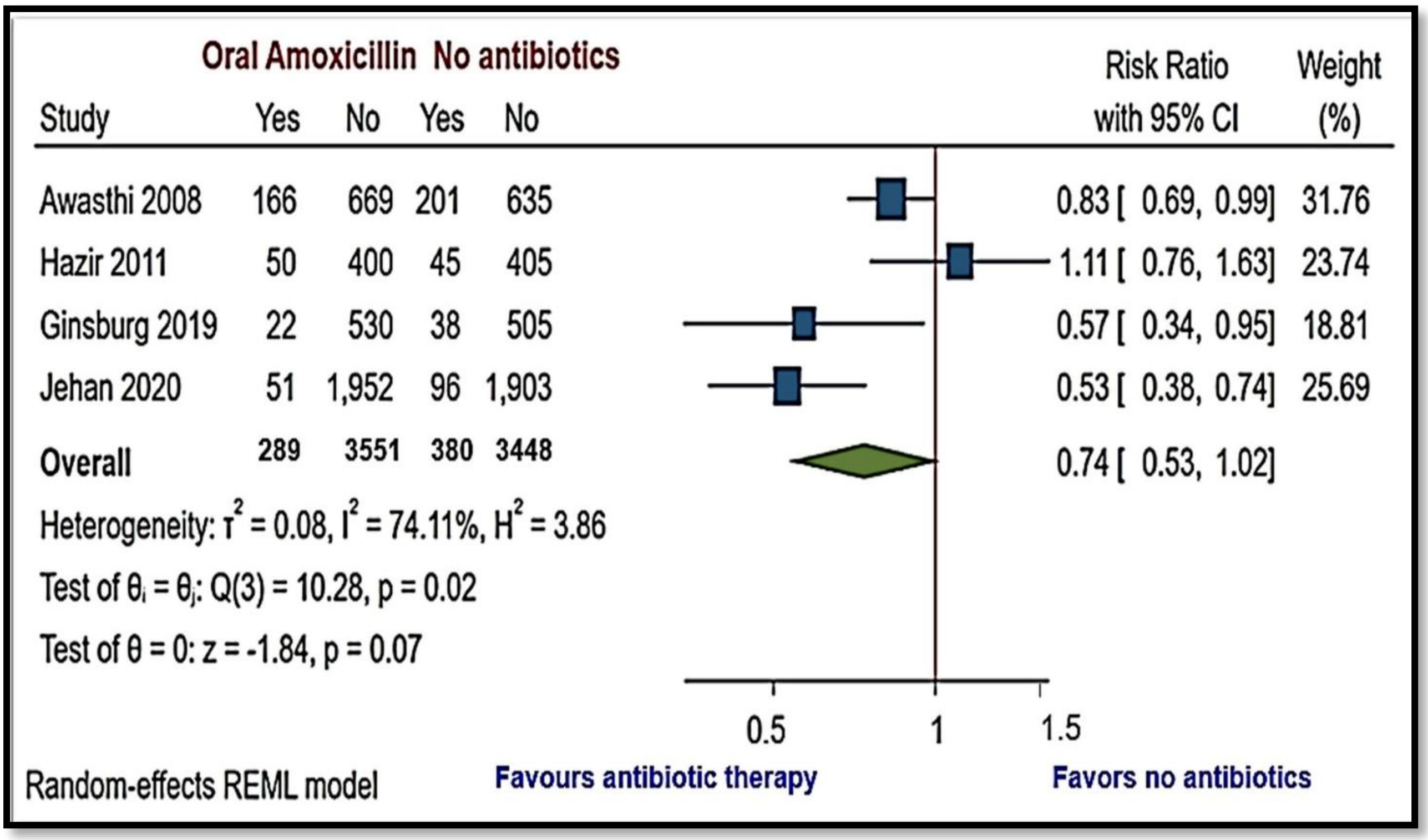

Supplementary Table 1: In children 2-59 months of age with only fast breathing, what is the effectiveness of oral amoxicillin compared to no antibiotic treatment in improving clinical outcomes.

Certainty assessment							№ of patients		Effect		Certainty	Importance
**№ of studies**	Study design	Risk of bias	Inconsistency	Indirectness	Imprecision	Other considerations	Oral amoxicillin	No antibiotics	Relative (95% CI)	Absolute (95% CI)		
**Treatment failure at day 4**												
**4**	randomized trials	serious[a]	not serious	not serious	serious[b]	none	288/3840 (7.5%)	379/3840 (9.9%)	RR 0.74 (0.53 to 1.02)	26 fewer per 1000 (from 46 fewer to 2 more)	⨁⨁◯◯ Low	CRITICAL
**Treatment failure at day 14**												
**4**	randomized trials	serious[a]	not serious	not serious	not serious	none	451/3840 (11.7%)	536/3828 (14.0%)	RR 0.84 (0.75 to 0.94)	22 fewer per 1000 (from 35 fewer to 8 fewer)	⨁⨁⨁◯ Moderate	CRITICAL
**Adverse events**												
**2[c]**	randomized trials	serious[a]	not serious	not serious	serious[b]	none	90/2567 (3.5%)	120/2561 (4.7%)	RR 1.08 (0.62 to 1.89)	4 more per 1000 (from 18 fewer to 42 more)	⨁⨁◯◯ Low	CRITICAL
**Adverse events (treatment initiation to 14 days)**												
**2[c]**	randomized trials	serious[a]	not serious	not serious	serious[b]	none	149/2567 (5.8%)	160/2561 (6.2%)	RR 0.93 (0.75 to 1.15)	4 fewer per 1000 (from 16 fewer to 9 more)	⨁⨁◯◯ Low	CRITICAL
**Mortality by day 14**												

**4**	randomized trials	serious [a]	not serious	not serious	serious [b]	none	1/3840 (0.0%)	1/3828 (0.0%)	RR 1.00 (0.17 to 5.75)	0 fewer per 1000 (from 0 fewer to 1 more)	⊕⊕◯◯ Low	CRITICAL

[a] downgraded one level as one study had high risk of bias.
[b] downgraded one level due to a wide confidence interval.
[C] Jehan et al. 2020 and Ginsburg et al.2019

Supplementary Table 2: In children 2-59 months of age with chest indrawing (without any danger signs), what is the effectiveness of oral amoxicillin on an outpatient basis compared to inpatient injectable antibiotics treatment in improving clinical outcomes.

**Certainty assessment**							**№ of patients**		**Effect**		**Certainty**	**Importance**
**№ of studies**	Study design	Risk of bias	Inconsistency	Indirectness	Imprecision	Other considerations	Oral amoxicillin	Injectable antibiotics	Relative (95% CI)	Absolute (95% CI)		
**Treatment failure at day 3**												
**3**	randomized trials	serious [a]	serious[b]	not serious	serious[c]	none	234/2172 (10.8%)	248/2157 (11.5%)	RR 0.88 (0.62 to 1.24)	14 fewer per 1000 (from 44 fewer to 28 more)	⊕◯◯◯ Very low	CRITICAL
**Treatment failure at day 6**												
**3**	randomized trials	serious [a]	not serious	not serious	serious[c]	none	304/2172 (14.0%)	314/2156 (14.6%)	RR 0.96 (0.83	6 fewer per 1000 (from	⊕⊕◯◯ Low	CRITICAL

									to 1.11)	25 fewer to 16 more)		
**Treatment failure at day 14**												
**3**	randomized trials	serious [a]	not serious	not serious	serious [c]	none	376/2153 (17.5%)	389/2143 (18.2%)	RR 0.94 (0.79 to 1.13)	11 fewer per 1000 (from 38 fewer to 24 more)	⨁⨁◯◯ Low	CRITICAL
**Mortality**												
**3**	randomized trials	serious [a]	not serious	not serious	serious [c]	none	4/2173 (0.2%)	14/2156 (0.6%)	RR 0.28 (0.09 to 0.86)	5 fewer per 1000 (from 6 fewer to 1 fewer)	⨁⨁◯◯ Low	CRITICAL
**Serious adverse events**												
**3**	randomized trials	serious [a]	not serious	not serious	serious [c]	none	3/2173 (0.1%)	12/2156 (0.6%)	RR 0.39 (0.12 to 1.26)	3 fewer per 1000 (from 5 fewer to 1 more)	⨁⨁◯◯ Low	CRITICAL

[a] downgraded one level as two studies had some concerns in risk of bias assessment.
[b] downgraded one level as I-square value 64.56% and P = 0.05.

[c] downgraded one level due to a wide CI.

Supplementary Figure 6: Oral Amoxicillin versus Injectable antibiotics: Adverse events among children aged 2-59 months with chest indrawing pneumonia on the 14th day from treatment initiation.

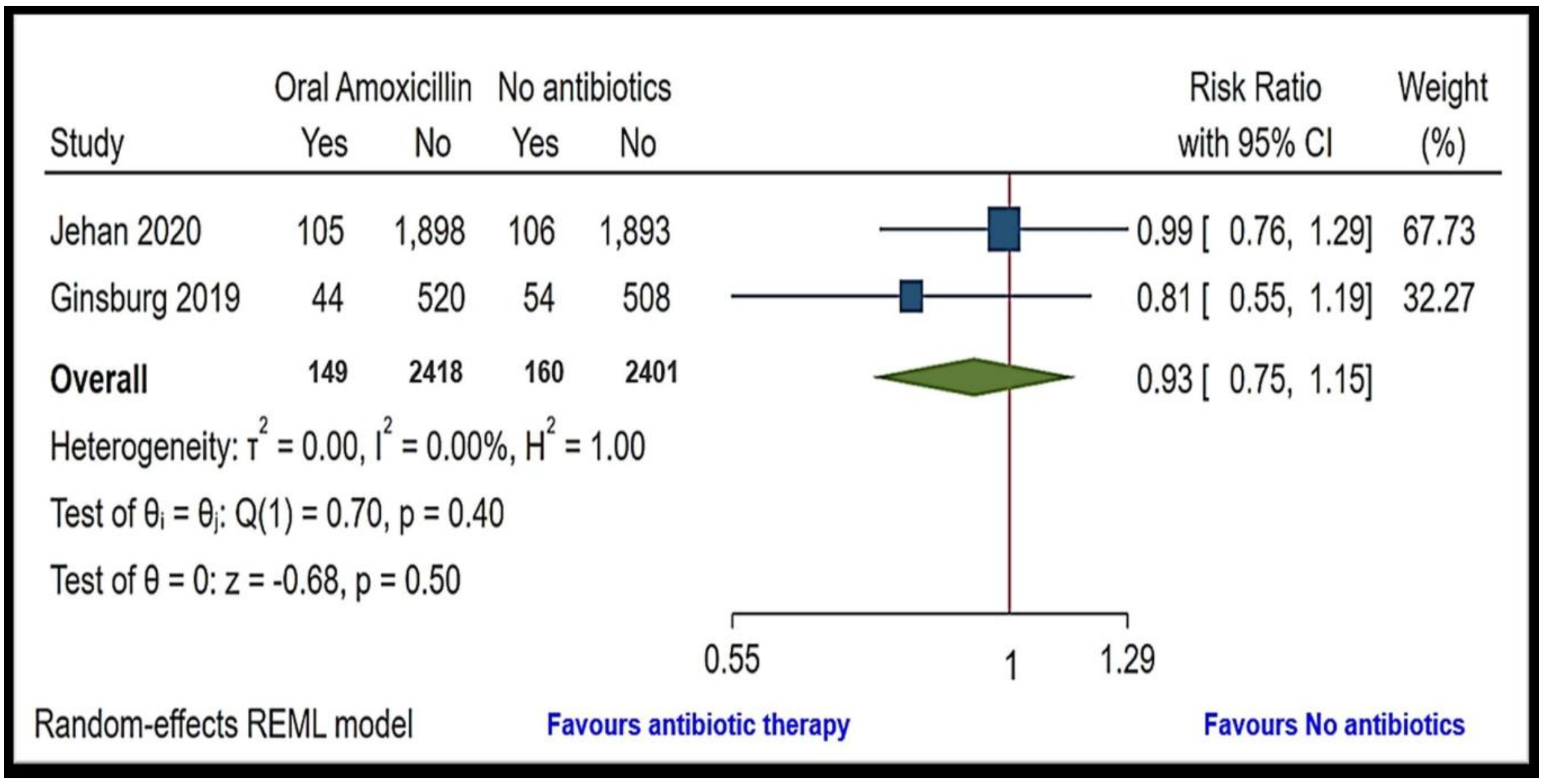

Supplementary Figure 7: Risk of all-cause mortality among the 2-59 months old children managed for fast breathing pneumonia

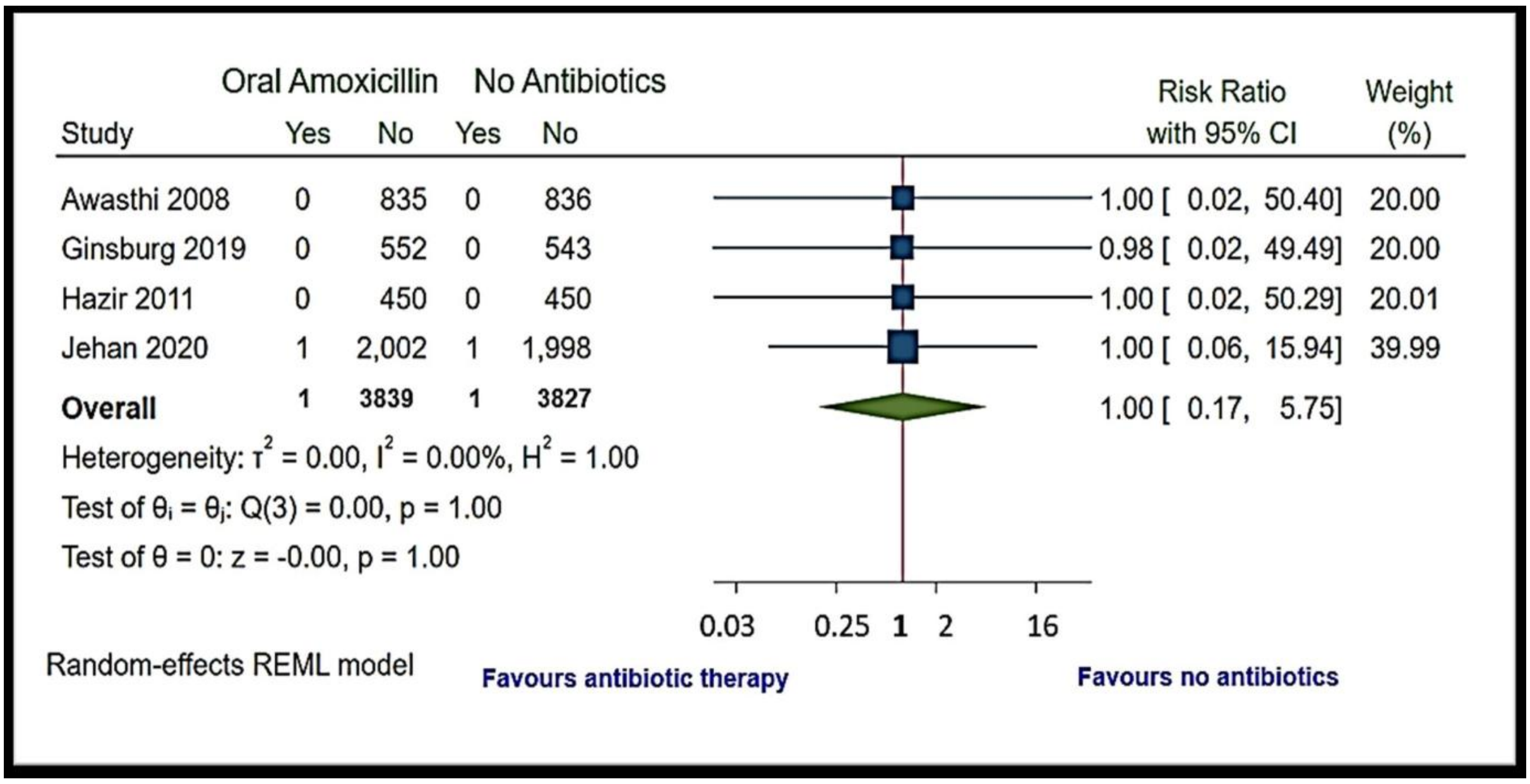

Supplementary Figure 8. Oral Amoxicillin versus Injectable antibiotics: Treatment failure among children aged 2-59 months with chest -indrawing pneumonia on 3rd day of management.

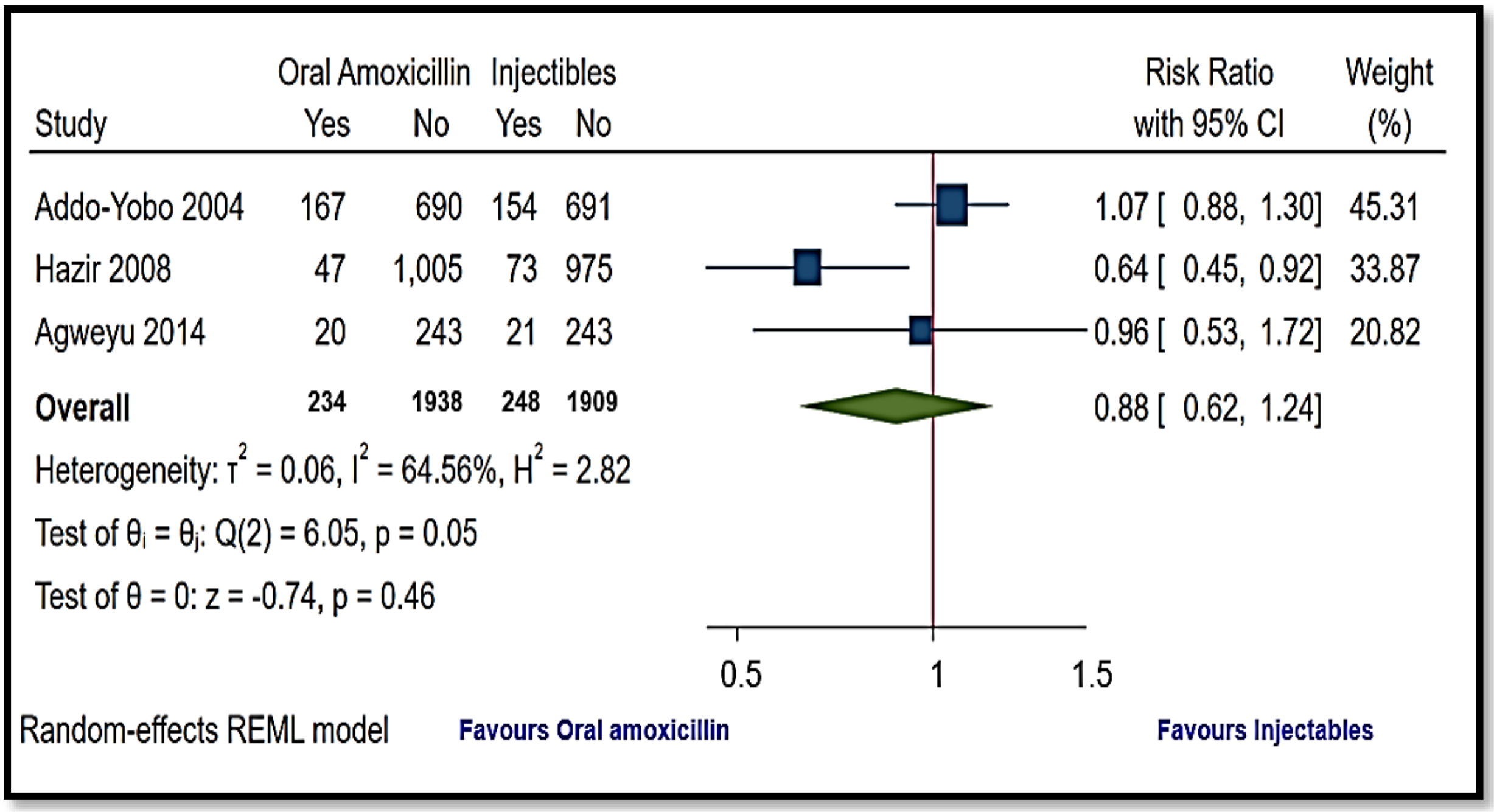

Supplementary Figure 9: Oral Amoxicillin versus Injectable antibiotics: Adverse events among children aged 2-59 months with chest indrawing pneumonia on the 14th day from treatment initiation.

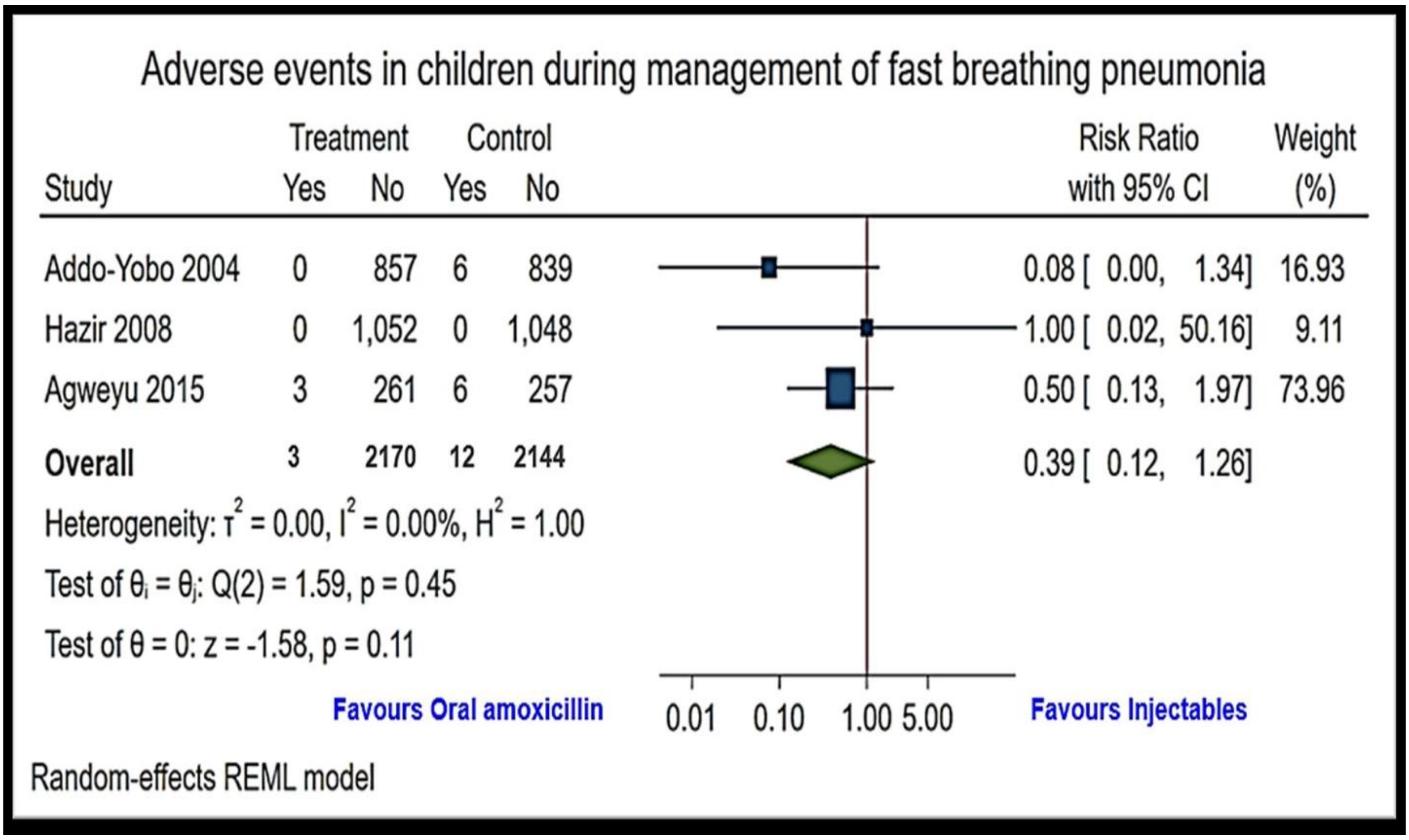

Supplementary Figure 10: Oral Amoxicillin versus Injectables antibiotics: All-cause mortality among children managed for chest indrawing pneumonia on the 14th day.

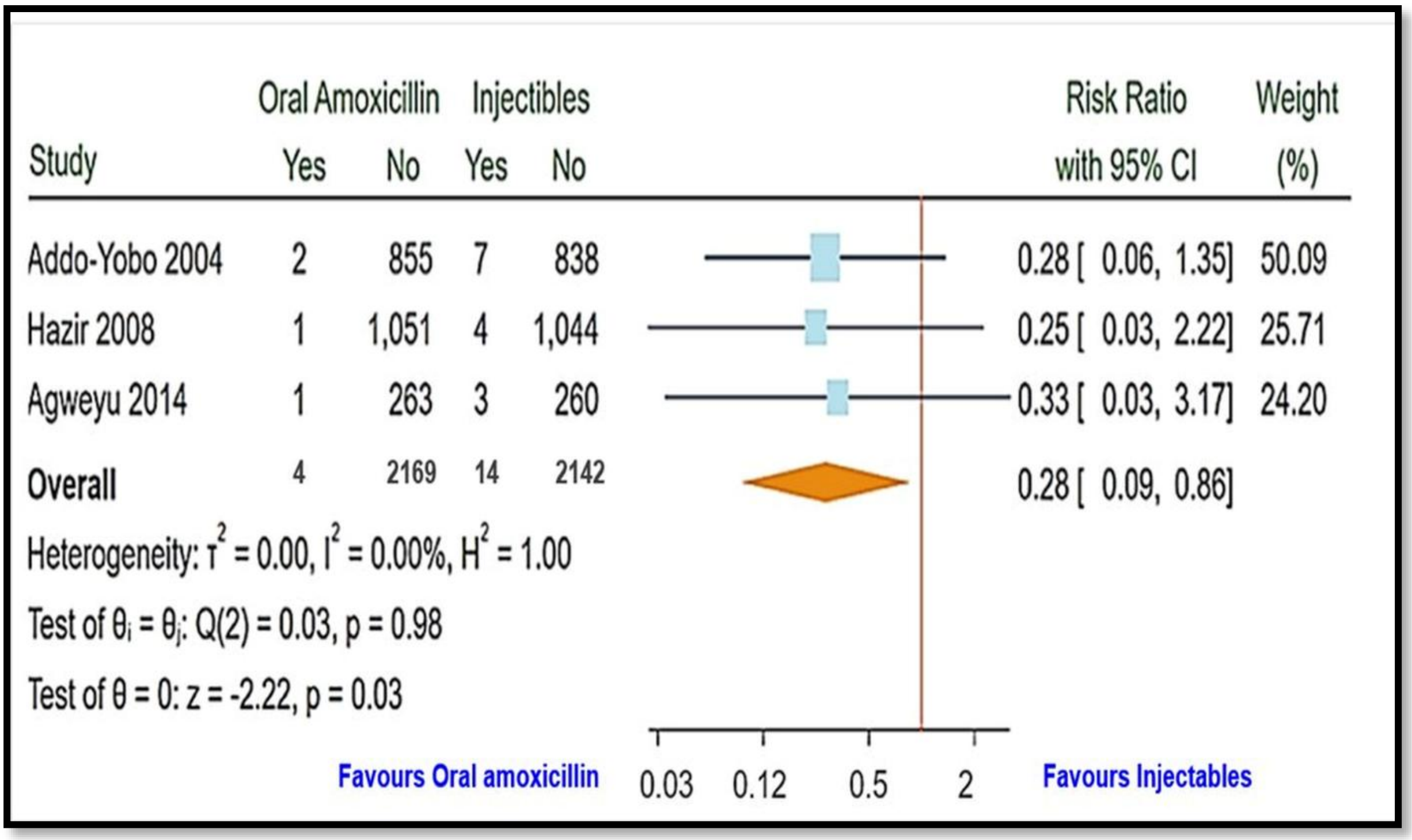

Supplementary Figure 11: Post hoc analysis following the roll-out of influenza vaccine: Risk of treatment failure in children (2-59 months) with fast breathing pneumonia assessed at 4 days

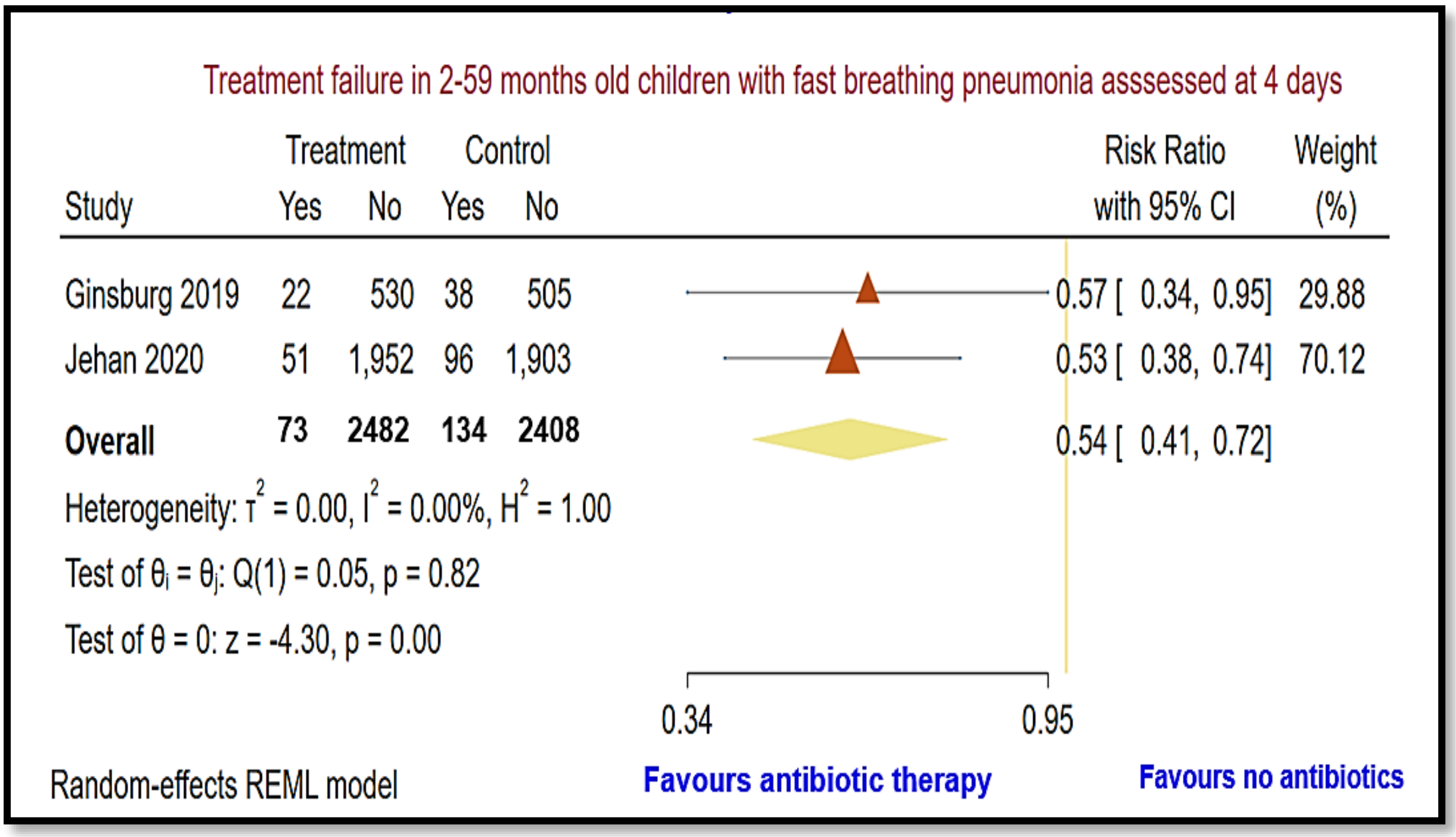

Supplementary Figure 11: Post hoc analysis following the roll-out of influenza vaccine: Risk of treatment failure in children (2-59 months) with fast breathing pneumonia assessed at 14 days

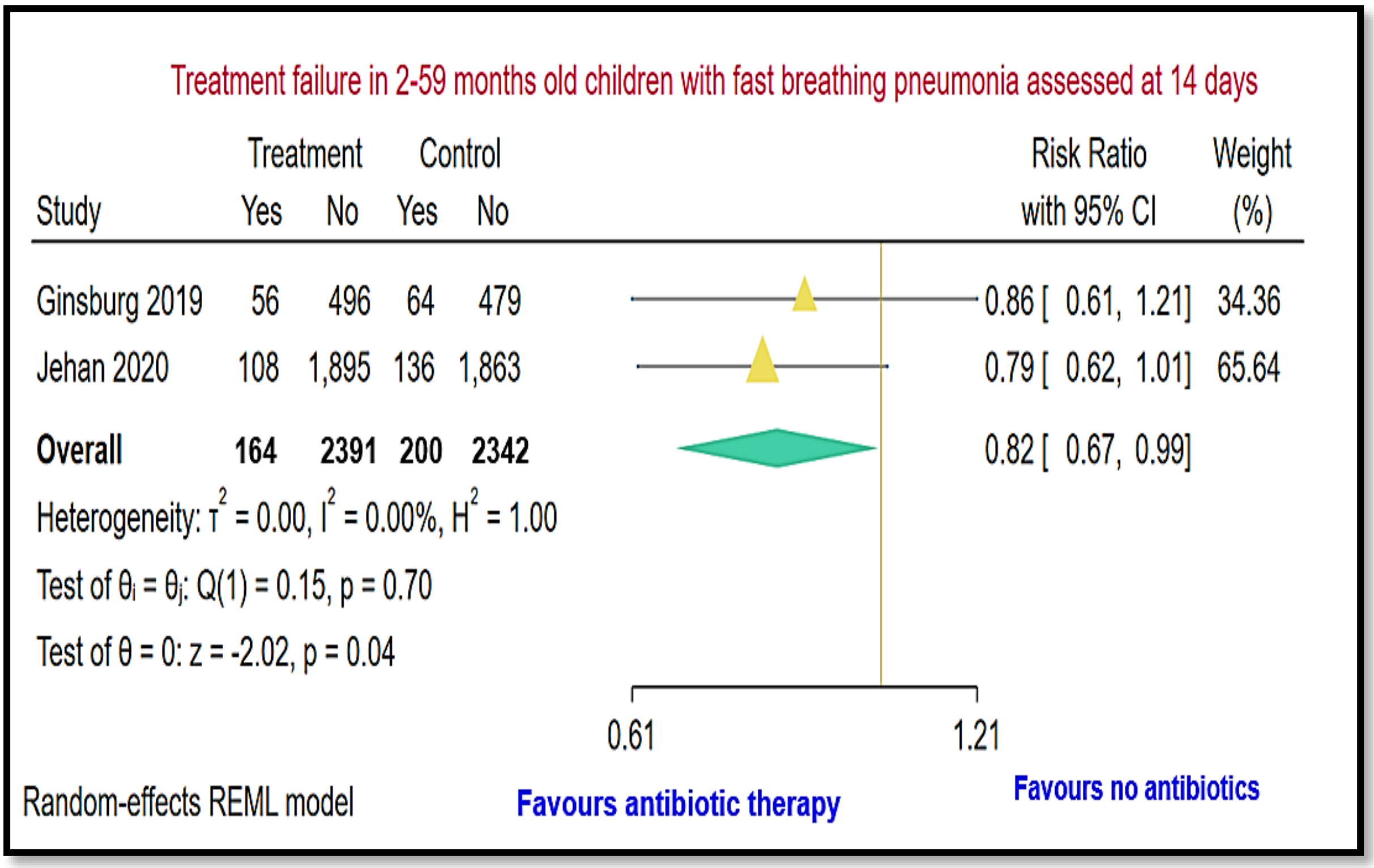

Operational definitions

**Box 2: Research question: Oral amoxicillin versus no antibiotics in managing fast breathing pneumonia among 2-59 months old children**	
**Study**	**Definition of clinical deterioration.**
Awasthi_2008	Presence of severe pneumonia or very severe disease (with or without wheeze) as defined by WHO (2000) Handbook of IMCI (Integrated Management of Childhood Illnesses.
Hazir_2011	Presence of lower chest indrawing or any of the WHO-defined general danger signs, specifically, lack of ability to drink
Ginsburg_2019	Presence of severe respiratory distress, Hypoxemia, WHO IMCI danger signs, Hospitalization due to pneumonia (if not initially admitted), Prolonged hospitalization or re-admission due to pneumonia (if initially admitted), Axillary temperature >38ºC in the absence of a diagnosed co-infection with fever symptoms (e.g., malaria)
Jehan_2020	Presence of WHO-defined danger signs, or retraction of the lower chest wall if the the patient was hospitalized.
Despite variations in operational definitions across studies, all definitions captured clinically relevant deterioration consistent with WHO IMCI criteria, supporting their conceptual comparability for pooled analysis.	

**Box 3: Research question: Oral amoxicillin versus injectables in managing chest indrawing pneumonia without danger signs among 2-59 months old children.**	
**Study**	**Definition of clinical deterioration**
Addo_Yobo 2004	Appearance of danger signs (Inability to drink, abnormal sleepiness, central cyanosis, convulsions), Low oxygen saturation (<80%), life-threatening or serious adverse drug reaction, newly diagnosed comorbid condition.
Agweyu_2014	Appearance of any danger signs (central cyanosis, head nodding, vomiting everything, inability to drink/breastfeed, grunting, oxygen saturation less than 85, prolonged hospitalization, and hospital readmission
Hazir_2008	Inability to take oral medication due to persistent vomiting, prolonged or hospitalization related to pneumonia; serious adverse events (development of danger signs- inability to drink, abnormal sleepiness, central cyanosis, convulsions)
Despite variations in operational definitions across studies, all definitions captured clinically relevant deterioration consistent with WHO IMCI criteria, supporting their conceptual comparability for pooled analysis.	
